# Fractal features of soil particle size distribution in newly formed wetlands in the Yellow River Delta

**DOI:** 10.1038/srep10540

**Published:** 2015-05-27

**Authors:** Junbao Yu, Xiaofei Lv, Ma Bin, Huifeng Wu, Siyao Du, Mo Zhou, Yanming Yang, Guangxuan Han

**Affiliations:** 1 Key Laboratory of Coastal Environmental Processes and Ecological Remediation, Yantai Institute of Coastal, Zone Research (YIC), Chinese Academy of Sciences (CAS); Shandong Provincial Key Laboratory of Coastal Environmental Processes, YICCAS, Yantai 264003; 2 University of Chinese Academy of Sciences, Beijing 100049, China; 3 College of Environmental Science and Engineering, Ocean University of China, Qingdao 266100, P. R. China; 4 Environment college, Northeast Normal University, Changchun 130024, P. R. China; 5 Cadre School of Science and Technology of Shandong Province, Ji’nan 250101, P. R. China

## Abstract

The characteristic of particle size distribution (PSD) in the newly formed wetlands in coast has seldom been studied. We applied fractal-scaling theory in assessing soil particle size distribution (PSD) features of newly formed wetlands in the Yellow River Delta (YRD), China. The singular fractal dimensions (*D*) values ranged from 1.82 to 1.90, the capacity dimension (*D*_0_) values ranged from 0.84 to 0.93, and the entropy dimension (*D*_1_) values ranged from 0.66 to 0.84. Constrained corresponding analysis revealed that 43.5% of the variance in soil PSD can be explained by environmental factors, including 14.7% by seasonal variation, 8.6% by soil depth, and 8.0% by vegetation type. The fractal dimensions *D* and *D*_1_ were sensitive with fine particles with size ranging less than 126 μm, and *D*_0_ was sensitive with coarse particles with size ranging between 126 μm to 2000 μm. Fractal analysis makes full use of soil PSD information, and offers a useful approach to quantify and assess the soil physical attributes in the newly formed wetland.

Coastal wetlands play important roles in the coastal ecosystems, offering wildlife food and habitat, as well as functioning as important nutrient cycling capacity for maintaining water quality^1^,^2^,^3^. Newly formed wetlands in estuary regions, where large quantities of silts have been deposited to form coastal wetlands, are generally found several hundreds of meters with vegetation changes gradually from barren land to halophytic vegetation and to hydrophyte vegetation^4^,^5^,^6^. The attribute changes in newly formed coastal wetlands can influence the ecosystem service functions of the coastal habitats.

Soil particle size distribution (PSD) is one of the most fundamental physical attributes due to its great influence on soil hydraulic characteristics, soil moisture movement, contaminant transport, and soil erosion^7^. Accordingly, characterizing variations of soil PSD is an important way to understanding and quantifying soil structure and dynamics^8^,^9^,^10^. The PSD characteristics in the newly formed wetlands are, however, still unclear.

Traditionally, textural triangle, the principal approach for soil PSD analysis, is restricted by the arbitrary definition of texture classes with incomplete information on the soil PSD^11^. Moreover, there is a high fluctuation of PSD in each textural class. A better approach is combining laser diffraction method and fractal analysis, which can highly promote the research of soil texture^10^,^12^,^13^,^14^,^15^. Fractal analysis offers the possibility of quantifying and integrating information on soil structure at different spatial scales^16^,^17^. Soil fractal analysis is currently implemented in two ways, i.e. singular fractal analysis and multifractal analysis^18^. Singular fractal analysis can be used to quantitatively describe soil PSD characteristics, soil aggregate fragmentation, and other related soil properties^16^. Multifractal analysis is mainly employed to capture the intrinsic variability of soil PSD to retain more detailed information^13^,^19^. Consequently, there are a number of studies applying multifractal analysis to study soil particle-size distributions^12^,^13^,^20^. Fractal theory is determined to the influence of different plant communities or land management on PSD^21^,^22^.

In this study, fractal scale theory was employed for assessing spatial and temporal variation of the PSD characteristics in a successional series of newly formed wetlands in the Yellow River Delta (YRD). The Yellow River is regarded as the largest contributor of fluvial sediment load to the ocean in the world^23^. The YRD is one of the most intensive land-ocean interaction regions among the large river deltas^4^, also has the youngest natural coastal wetland ecosystem in China. The objectives of this work were to 1) analyze the PSD and the fractal dimensions of soils in the newly formed wetlands of the YRD, and 2) explore the impacts of vegetation community on the spatial and temporal variation of soil PSD in the newly formed wetlands.

## Methods

### Study area

**Figure 1:**
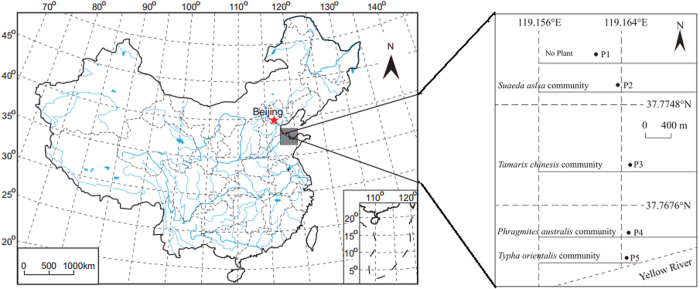
The location of study region and sampling sites (ARCGIS 9.3).

The investigation was conducted at the YRD located in northern part of Shandong Province, China. The YRD (118.6°E-119.3°E, 37.6°N-38.2°N) is characterized as a temperature, semi-humid continental monsoon climate. The average temperature is 11.7–12.6 °C, and the average annual evaporation is 1900–2400 mm. The average annual precipitation is 530–630 mm, of which 70% is rainfall during June to September (in summer and fall). The soil is typical saline alluvial soil (Fluvisols, FAO). The net increase of delta shoreline length was ~61.64 km with annual increase of ~1.81 km, and net extension of newly formed wetland area was ~309.81 km^2^ with rate of ~9.11 km^2^ year^−1^ in the YRD^24^. The coastal wetlands in the YRD have clear horizontal distribution vegetation zones of ecosystems with the changes in soil salinity from seaside to inland (Fig. 1). A total of 400 plant species are recorded in the region, of which 55.1% vegetation is natural saline vegetation^25^. The dominant plant species adapt the saline-alkline are *Tamarix chinensis*, *Suaeda salsa* and *Phragmites australis* in the YRD.

### Sampling and processing

We chose five sampling plots along the bank of the Yellow River in a successional series of newly formed wetlands (formed since 1976) based on vegetation community. The dominant plant species in sampling plots were no plant (P1), *Suaeda aslsa* (P2), *Tamarix chinesis* (P3), *Phragmites australis* (P4) and *Typha orientalis* (P5), respectively (Fig. 1). Soil samples were collected from five random soil cores and 0–10, 10–20, 20–30 cm depth with in wet season (July, October 2012), and dry season (February, May 2013). The replicated samples were mixed homogeneously at each location to form a composite sample. We collected 60 samples totally in this study for the following analysis.

The visible roots and rocks were removed prior to further processing. Soil samples were air-dried and passed 2 mm sieve. After dispersed by sodium hexametaphosphate (NaHMP) and ultrasonic for 30 s, the samples were analyzed with Longbench Mastersizer2000 (Malvern Instruments, Malvern, England) to get particle size distribution ranging from 0.2 μm to 2000 μm. The interval of particle sizes (μm) *I *= [0.2, 2000] were graded into 64 subintervals *I*_*i*_=[ø_*i*_, ø_*i* +1_], *i*=1, 2, ···, 64, and the lengths of subintervals followed a logarithmic scale such that log(ø_i+1_/ø_i_) is constant. Meanwhile, according to the United States Department of Agriculture classification of soil particle size, the soil size was partitioned into 3 grades, clay (0–2 μm), silt (2–50 μm) and sand (50–2000 μm). The soil organic matter (SOM) was determined by K_2_CrO_7_ routine colorimetric method^26^.

### The singular fractal analysis for soil PSD

The singular fractal dimension (*D*) of soil PSD was estimated from the following equation^27^:


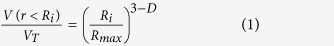


Where *V*(*r* < *R*_*i*_) is the cumulative percentage of particles of *i* size *r* less than *R*_*i*_, *V*_*T*_ is the total percentage (*V*_*T*_ = 100), *R*_*i*_ is the particle radius (mm) of the *i* size class, and *R*_*max*_ is the radius of the largest particle class (*R*_*max*_ = 1, in this study). The particle diameter is taken as the upper sieve sizes. Taking logarithms on both sides of Equation (1), the *D* value can be derived by the slopes of the logarithmic linear regression equation.

### The multifractal analysis for soil PSD

The multifractal dimension (Rényi dimension) was calculated using Equation (2) and Equation (3) in this study. A number of cells with size of *ε* to cover the entire interval, and the cell number is *N*, the Rényi dimension is computed by the mass of soil particles in subinterval *μ*_*i*_(*ε*), cell diameter *ε* and the parameter *q*^12^.


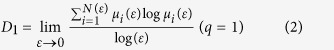



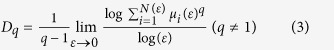


*D*_*q*_ extracts the system parameters from different levels with the *q* value in the interval [∞, +∞]. The Rényi dimensions for *q *= 0 and *q* = 1 are known as capacity dimension *D*_0_ and entropy dimension *D*_1_, respectively. In multifractal systems, *D*_0_ is the capacity dimension is known as box-counting dimension. It provides average information of PSD system and reflects the range of a continuous distribution. In this study, all the PSDs are distributed continuously from 0.3 μm to 2000 μm. So *D*_0_ = 1 means the interval of particle sizes were all occupied by all scales. The PSDs with low *D*_0_ hold a narrow range. The entropy dimension, *D*_1_, provides a measure of the heterogeneity of a PSD. The higher the value of *D*_1_ is, the more heterogeneous the soil’s PSD is, and the wider the range of PSD is. Considering that *D*_0_ provides general information and *D*_1_ measures the homogeneity of PSD system, *D*_1_/*D*_0_ is used to describe the heterogeneity in a distribution to obtain the relation between the two parameters. As *D*_1_ takes values less than *D*_0_, the quotient *D*_1_/*D*_0_ is less than 1. The closer to 1 of *D*_1_/*D*_0_ refers the more evenly dispersed in the fractions over the set of sizes, and the more heterogeneous in the distribution.

### Statistical analysis

Data in the figures and tables were mean values of each sample. All statistical analyses were implemented using various packages within the R statistical computing environment. One-way analysis of variance (ANOVA) procedures were used to detect the differences in measured parameters among plots. Constrained correspondence analysis (CCA) was used to assess the relationship between soil PSD profiles and environmental variables with the *vegan* package.

Network analyses can be used to show the composition of, and interactions between, multiple elements in communities. A matrix of correlation between all trait pairs was generated. Significant levels were set at *p* < 0.05. The total significant pairs were considered as a network in which a vertex corresponds to a trait and a link between two vertices corresponds to significant correlations. This network plot was then subjected based on the adjacency matrix with *igraph* package. Essentially, this algorithm divides the network into modules or groups of vertices that are more connected between themselves than to nodes from other modules, yielding a cartographic representation of a complex network.

## Results

### Soil particle size distribution

**Figure 2:**
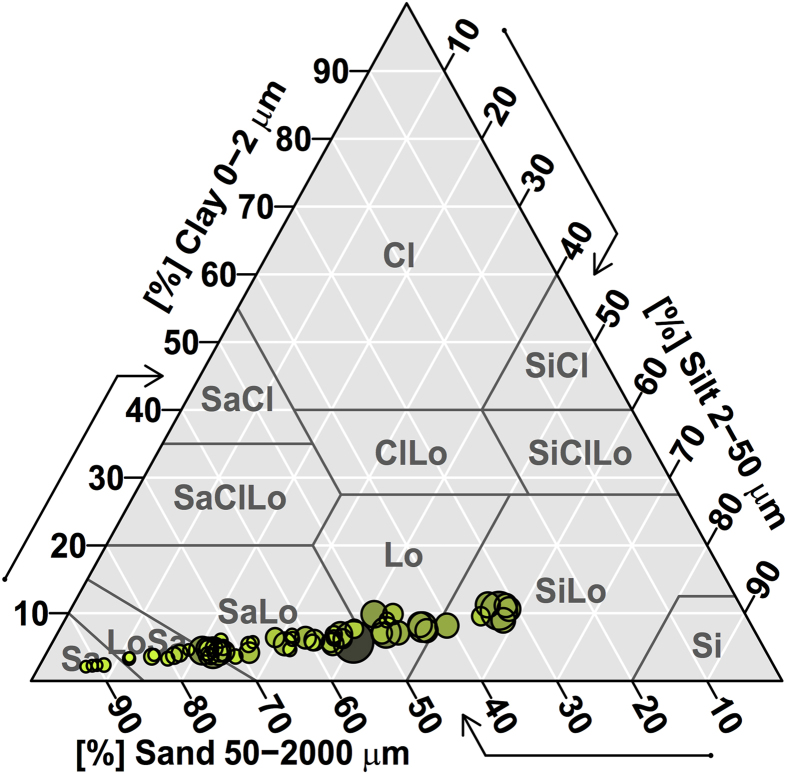
Texture of analyzed soil samples. The size of plots indicates the concentrations of soil organic matter.

**Figure 3:**
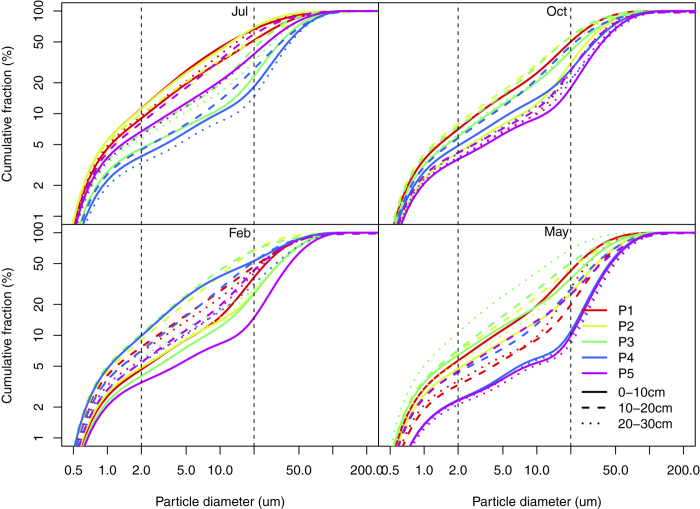
Log-long plots of particle size distributions for soil samples.

The soil texture changed from sand to silt loam with the soil organic matter concentration increasing (Fig. 2). The predominant soil particle was sand fraction, accounting for 62% of total soil volume (Fig. 3). The proportion of silt and clay particles was 31% and 6%, respectively.

The sand fraction in P4 (*Phragmites australis*) and P5 (*Typha orientalis*) were greater than those in P1 (no plant), P2 (*Suaeda aslsa*) and P3 (*Tamarix chinesis*) (Table 1). The proportions of sand in topsoil layers (0–10 cm) were smaller than those in underlying soil layers (10–30 cm). The seasonal variations of the clay and sand fractions were significant, but did not appeared in the silt fraction. The fine particles (clay and silt) proportion was the highest in May and the lowest in July.

### Singular fractal dimension characteristics of soil PSD

The singular fractal dimension (*D*) values ranged from 1.82 to 1.90 (Table 1). Analysis of variance showed the significant differences in the singular fractal dimensions in different vegetation and soil layers. The *D* values of P2 and P3 were higher than those of other areas (P1, P4 and P5). The *D* values in the topsoil were higher than those in the underlying soil layers.

### Multifractal characterization of soil PSDs

**Figure 4:**
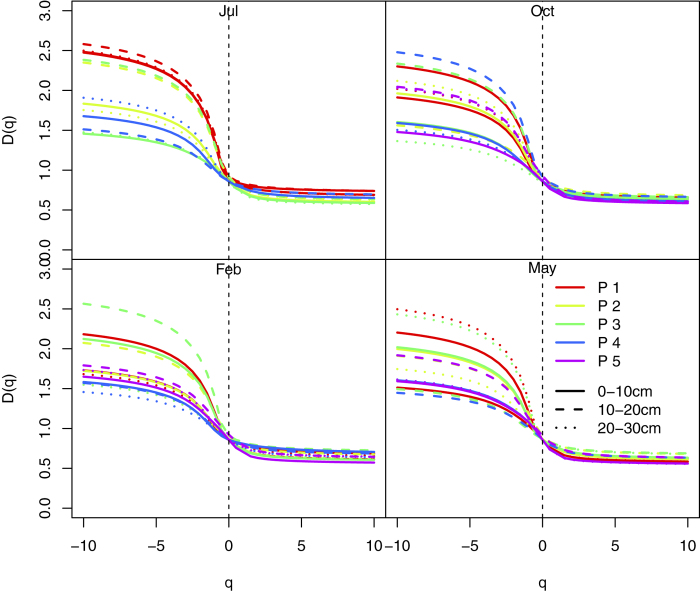
The Rényi dimensions spectra for soil samples.

The values of capacity dimension (*D*_0_) and entropy dimension (*D*_1_) varied from 0.84 to 0.93 and 0.66 to 0.84, respectively. The values of *D*_1_/*D*_0_ ranged from 0.76 to 0.94 (Fig. 4, Table 1). The multifractal dimensions were significantly different in different vegetation. The *D*_0_ values were the highest in P3 and P4. The highest and the smallest values of *D*_1_ appeared in P4 and P3, respectively. The *D*_1_/*D*_0_ values in P1, P2, and P4 were greater than those in P3 and P5 (Table 1). Soil layers significantly influenced *D*_0_ and *D*_1_, but did not affect *D*_1_/*D*_0_. The values of multifractal dimensions in 0–10 cm layer were greater than in underlying soils layers. The values of *D*_1_ and *D*_1_/*D*_0_ were significantly different in different seasons (Table 1). The *D*_1_ values and the *D*_1_/*D*_0_ values in July were lower than in other seasons. The *D*_1_ and *D*_1_/*D*_0_ values in February and May were higher than those in October and February (Table 1).

### Network analysis and CCA analysis

**Figure 5:**
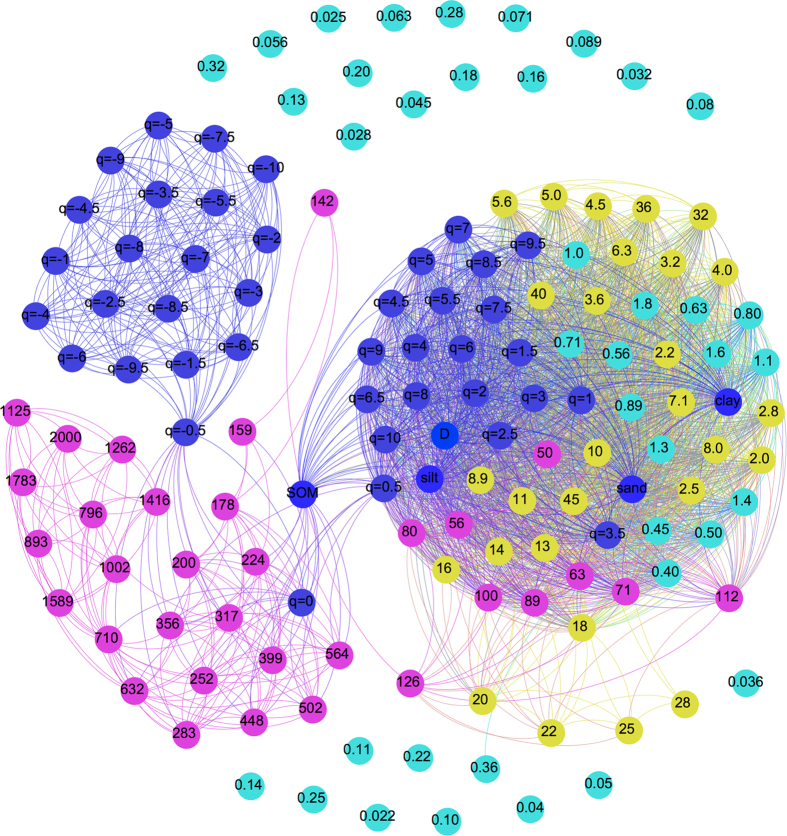
Network shows the associations between soil particles, fractal parameters and soil organic matter (SOM). The lines indicate significant correlations (*p* < 0.05, spearman correlation index). The blue nodes represent fractal dimensions and soil properties. The pink nodes represent sand particles, the yellow nodes represent silt particles, and the green nodes represent silt particles. The numbers in the nodes represent the size of soil particles.

To test the association relationships between soil PSD and fractal dimensions, a combinatorial soil PSD-*D*_*q*_ network was constructed (Fig. 5). All significant correlations (*p* < 0.05) were visualized as edges. The figures in the dots mean the size (μm) of the soil particles. The resulting network contained three modules: one was large, strongly connected, and the other two were small weakly connected. The large and interconnected module contained soil size grades (clay, silt and sand) and soil particle with the size ranging from 0.40 μm to 126 μm. The *D* and Rényi dimension spectra (*D*_q_) for *q *> 0 were also included. One of the small loosely interconnected modules contained the capacity dimension *D*_0_ and soil particle size ranging 126–2000 μm. The other small loosely connected module contained all of *D*_q_ for q < −0.5. The SOM connected the large module and the soil particle with size ranging between 224 μm and 564 μm.

The CCA model revealed that 43.5% of the variance in PSD could be explained by environmental factors, including 14.7% for seasonal variation, 8.6% for soil depth and 8.0% for vegetation difference. The variation of PSD was significantly correlated with the seasonal variation (R^2^ = 0.147 and *p* = 0.009) and the soil PSD were related with the depth (R^2^  = 0.086, *p*  = 0.047). The significant relation between PSD and vegetation was not observed in the study (R^2^  = 0.080, *p* = 0.06).

## Discussions

The range of *D* values was relatively low (1.82–1.90) in newly formed wetland of the YRD. In soils with high fertility and fine texture, the *D* values ranged from 2.60–2.80, however the *D* values ranged from 1.83–2.64 in soil with poor structure and coarse texture^19^,^28^,^29^. Accordingly, the soils in newly formed wetlands of the YRD were relatively coarse and infertility. Although the abundance of plant is great in this area^25^, the soil still has poor quality because the formation of the newly formed wetland is less than 40 years (since 1976). The sand fractions in soils near the riverbank were markedly greater than those in other plots. This result was similar to the results in mangrove forests and coast, where the sand fractions are higher in soils near the seawater^30^. The water flow could transport fine particles in soils much easier, leading to coarse texture in soils near the river or seawater.

The fractal dimensions *D* and *D*_*1*_ were highly correlated with particles less than 126 μm and SOM contents. The fractal dimensions *D*_0_ is correlated with coarse particle ranging from 126 μm to 2000 μm. Liu *et al.*^29^ found the significant correlation between singular fractal dimension and clay proportion. Wang *et al.*^12^ confirmed that *D*_1_ and *D*_1_/*D*_0_ were significantly positively correlated with fine particle content and SOM. Other studies have also obtained similar results that the high *D* values were observed in the soil with fine texture in different landscapes under contrasting climate conditions^28^,^30^,^31^,^32^. However, the range of particle size correlated with *D* and *D*_1_ values was not declared in former studies. Using networks analysis, we found that the size of particles correlated with *D* and *D*_1_ values were less than 126 μm. In pedogenic processes, soil organic carbon and nutrients accumulation, soil structure development and some biological properties are accompanied by selective increasing in fine particle size fractions^14^,^18^. The smaller particle sizes reflect the greater the spatial filling capacity of the soil corresponding higher fractal dimension values based on pore geometry^29^. Accordingly, fractal dimensions (*D*_1_, *D*) that mainly represent fine soil particles are high correlated with SOM content in soils. The fractal dimension *D*_0_ can prove useful information about the coarse particles, which are neglected in fractal dimensions *D* and *D*_1_ values.

The variation of soil PSD from newly formed wetlands in the YRD was influenced greatly by seasonal variation, soil depth, and plant vegetation. We found that the fractal dimension in the dry season (February and May) was greater than in the wet season (July and October). The fractal dimension in dry season was found to be lower than in wet season because the aggregates were easily dispersed in wet condition^33^. However, the clay particles could significantly decrease with increasing rainfall intensity since the fine particles were easily detached and transported by water (rainfall)^34^. Furthermore, the stronger plant root actives in wet season can increase fractal dimension through increasing soil organic matters, which is essential for aggregate formation^31^.

The fractal dimensions in topsoil were higher than that in sub-layer soils because of well-developed structure and easily dispersed soil particle (fine particle) in topsoil. High fractal dimension on surface soil is associated with fine texture and high fertility^18^,^28^. The soil organic matters and nitrogen concentrations are positively correlated with fractal dimensions^28^. Accordingly, the higher soil organic matters and nitrogen concentrations in topsoil layers would result in greater fractal dimensions. The incidence of vegetation root activities could increase the soil fractal dimension^35^, because the plant root activities could significantly increase topsoil aggregate stability^36^.

The fractal dimension values in soils with vegetation were significantly higher than those in soils without plant covering. Our results agree with other studies that soils with plants have higher *D* values^12^,^28^,^29^. Vegetation coverage can increase the ratio of fine particles, improve the soil structure, and decrease the risk of soil wind erosion^37^,^38^. The fractal dimensions in soils under various plant species are different. The root structures of various plant species might be a source of fractal dimensions difference^39^.

## Conclusions

The fractal dimensions were low in a newly formed wetland in the YRD. Different fractal dimensions of PSD were influenced by soil particles with different sizes. The fractal dimensions *D* and *D*_1_ were sensitive with fine particles (<126 μm) and the fractal dimension *D*_0_ was sensitive with coarse particles (126–2000 μm). The fractal dimensions were greater in wet season than in dry season. This result indicated that the influence of organic matters in soils was greater than that of soil water content. The fractal dimensions in topsoil layers were greater than subsoil layers because of the influence of root activities. Moreover, the fractal dimensions in soils with various plant species were obviously different and were greater than in soils without vegetation. Consequently, the soil particle size distribution is greatly affected by plant root activity in newly formed wetlands in the YRD. Vegetation management is the most effectively approach for increasing soil quality. Fractal analysis makes full use of soil PSD information, and offers a useful approach to quantify and assess the soil physical attributes in newly formed wetland.

## Additional Information

**How to cite this article**: Yu, J. *et al.* Fractal features of soil particle size distribution in the newly formed wetlands of the Yellow River Delta estuary. *Sci. Rep.* **5**, 10540; doi: 10.1038/srep10540 (2015).

## Figures and Tables

**Table 1:** Effect of vegetation, soil depth and seasonal variation on singular fractal dimensions (*D*), capacity dimension (*D*_0_), entropy dimension (*D*_1_), *D*_1_/*D*_0_, soil texture in soil.

**Factor**	**Variation**	**D**	**D**_**0**_	**D**_**1**_	**D_1_/D_0_**	**clay**	**Silt**	**Sand**
Vegetation	P1	1.84	0.87	0.76	0.87	6.55	36.58	56.87
	P2	1.88	0.86	0.76	0.88	6.78	36.37	56.85
	P3	1.87	0.88	0.74	0.84	6.73	36.39	56.88
	P4	1.85	0.88	0.77	0.88	4.80	23.54	71.66
	P5	1.85	0.87	0.74	0.85	4.67	23.81	71.51
	F values	2.95	2.95	2.51	3.08	3.82	4.32	4.34
	*p* values	0.03	0.03	0.05	0.02	0.01	0.01	0.01
	0–10	1.90	0.89	0.78	0.88	6.87	38.97	54.15
Soil depth	10–20	1.84	0.86	0.74	0.86	5.39	28.1	66.51
	20–30	1.83	0.86	0.74	0.86	5.46	26.94	67.59
	F values	22.36	22.36	12.45	1.715	3.85	6.48	6.19
	*p* values	0	0	0	0.19	0.03	0.01	0.01
	Jul(2012)	1.84	0.87	0.73	0.84	4.77	24.92	70.3
Seasonal variation	Oct(2012)	1.86	0.87	0.75	0.86	5.28	30.13	64.58
	Feb(2013)	1.85	0.87	0.76	0.88	6.35	36.39	59.67
	May(2013)	1.87	0.88	0.77	0.88	7.22	36.32	56.45
	F values	1.8	1.8	5.36	3.17	4.98	2.74	3.05
	*p* values	0.16	0.16	0.002	0.03	0.01	0.05	0.04
